# A Peculiar New Pampatheriidae (Mammalia: Xenarthra: Cingulata) from the Pleistocene of Argentina and Comments on Pampatheriidae Diversity

**DOI:** 10.1371/journal.pone.0128296

**Published:** 2015-06-17

**Authors:** Flávio Góis, Laureano Raúl González Ruiz, Gustavo Juan Scillato-Yané, Esteban Soibelzon

**Affiliations:** 1 Laboratorio de Paleontología de Vertebrados, Centro de Investigaciones Científicas y Transferencia de Tecnología a la Producción (CICYTTP–CONICET), Materi y España, 3105 Diamante, Entre Ríos, Argentina; 2 Laboratorio de Investigaciones en Evolución y Biodiversidad (LIEB), Universidad Nacional de la Patagonia ‘San Juan Bosco’ sede Esquel (UNPSJB), Ruta Nacional 259, km 16.5, 9200, Esquel, Chubut, Argentina; 3 División Paleontología Vertebrados, Museo de La Plata, Paseo del Bosque, s/n, 1900, La Plata, Buenos Aires, Argentina; 4 CONICET, Consejo Nacional de Investigaciones Científicas y Técnicas, Rivadavia, 1917, Ciudad Autónoma de Buenos Aires, Buenos Aires, Argentina; New York Institute of Technology College of Osteopathic Medicine, UNITED STATES

## Abstract

Pampatheriidae are a group of cingulates native to South American that are known from the middle Miocene to the lower Holocene. Two genera have been recognized between the lower Pleistocene and the lower Holocene: *Pampatherium* Gervais and Ameghino (Ensenadan, Bonaerian and Lujanian, lower Pleistocene–lower Holocene) and *Holmesina* Simpson (Blancan, Irvingtonian, upper Pliocene–lower Holocene). They have been mainly differentiated by their osteoderm morphology and cranio-dental characters. These taxa had a wide latitudinal distribution, extending from the southern part of South America (Península Valdés, Argentina) to North America (Florida, USA). In this contribution, we describe a new genus and species of Pampatheriidae for the lower and middle Pleistocene of Buenos Aires Province and for the upper Pleistocene of Santa Fe Province (Argentina).The new taxon is represented by disarticulated osteoderms, one skull element, two thoracic vertebrae and a right femur and patella. It has extremely complex osteoderm ornamentations and particular morphological characters of the cranial element and femur that are not found in any other species of the family. This new taxon, recorded in the lower–middle Pleistocene (Ensenadan Stage/Age) and in the upper Pleistocene–early Holocene (Lujanian Stage/Age), is incorporated to the Pleistocene mammal assemblage of South America. Finally, the Pampatheriidae diversity is greater during the Lujanian Stage/Age than the Ensenadan Stage/Age.

## Introduction

Xenarthra forms a clade of mammals widely distributed in the Americas. The earliest record of a Xenarthra is precisely a Cingulata (Dasypodidae) described for the upper Paleocene (Itaboraian SALMA) [1, 2, 3, 4] or early Eocene [5] of Brazil, while in Argentina they are represented for the lower Eocene (Riochican SALMA) [6, 7, 8]. During the Paleogene, Neogene and Quaternary, the cingulates showed a greater diversity than today, including taxa with a wide range of sizes and habitats [6, 9, 10, 11].

A remarkable feature of all cingulates is the presence of a bony caparace covering most of the skull, the back and the tail (except in *Cabassous* McMurtrie, 1835). This dorsal carapace consists of individual pieces called osteoderms, which are covered by horny scales (of epidermal origin). Additionally, osteoderms can be found in the integument of the face, in the ventral region of the body and in the limbs, though without forming a continuous shield [12, 13, 14, 15, 16, 17, 18, 19].

The Cingulata include living and extinct forms grouped into two superfamilies: a) Dasypodoidea, with Peltephilidae and Dasypodidae families and b) Glyptodontoidea, with three main families, Glyptodontidae, Pampatheriidae and Palaeopeltidae [20].

The recognition of Pampatheriidae as dasypodoids or glyptodontoids has been discussed as they have morphological characters that can be associated alternately with either group. They should be included within Dasypodoidea especially for having the caparace divided into three regions (scapular buckler, movable bands and pelvic buckler), the anatomy of the limbs and certain cranial characters (e.g., development of the snout) [21, 22, 23, 24, 25, 26, 27]. But, they resemble Glyptodontoidea in the caparace morphology (globular aspect, size and thickness of osteoderms), characters of the auditory region (petrosal with a narrow and triangular promontory), some cranial robustness and the morphology and function of the masticatory apparatus, the ascending ramus of the mandible anteriorly inclined, the elevation of the basicranial axis relative to the palate, and the elevation of the mandibular notch well above the dental series [16, 28, 29, 9, 30, 31, 32].

The pampatheres are recorded for the first time in South America in the middle Miocene [33], but see [34] and their last record was in the lower Holocene [35, 31, 32]. Prior to this work six genera were recognized, four of them were recorded during the Neogene (*Scirrotherium* Edmund and Theodor, 1997, *Kraglievichia* Castellanos, 1927, *Vassallia* Castellanos, 1927 and *Plaina* Castellanos, 1937) whereas the other two were recorded during the Quaternary (*Pampatherium* Gervais and Ameghino, 1880 and *Holmesina* Simpson, 1930) [33, 36, 37, 38, 14, 15, 39, 40, 35, 30, 31, 32].

The material that motivates this manuscript was originally assigned to *Pampatherium typum* Gervais and Ameghino, 1880 [38] and subsequently included in general Pleistocene faunal lists (Ensenadan) [41, 42, 43], however a detailed study of the material allows us to identify a new taxon [32, 44].

**Fig 1:**
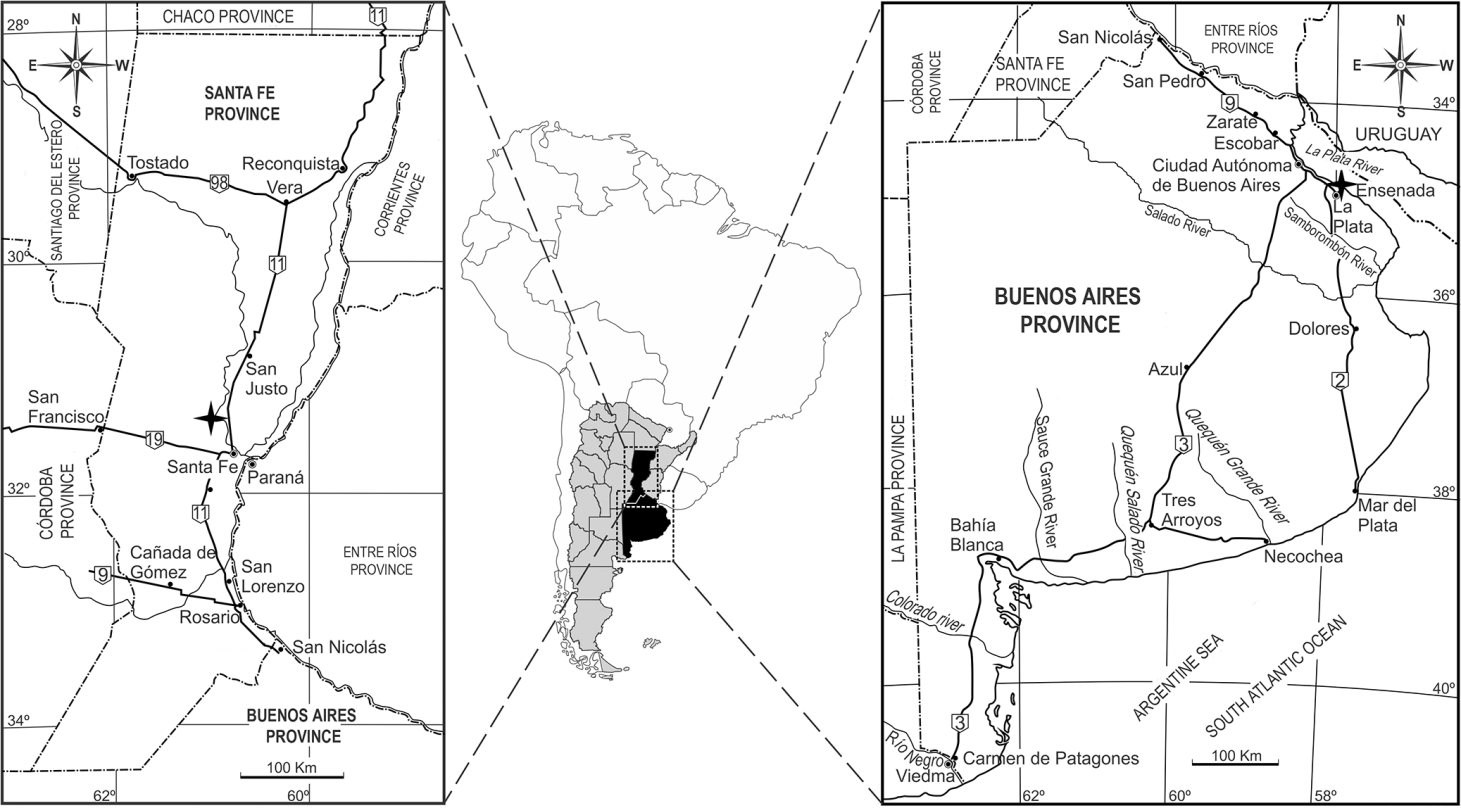
Geographic location of fossil localities mentioned in the text.

Therefore, the aim of this paper is to present this peculiar new genus and species of Pampatheriidae for the Pleistocene (Ensenadan Stage/Age and Lujanian Stage/Age) of Buenos Aires and Santa Fe provinces of Argentina (Fig 1) with comments on the diversity of Pampatheriidae during this period.

## Stratigraphic Context

**Fig 2:**
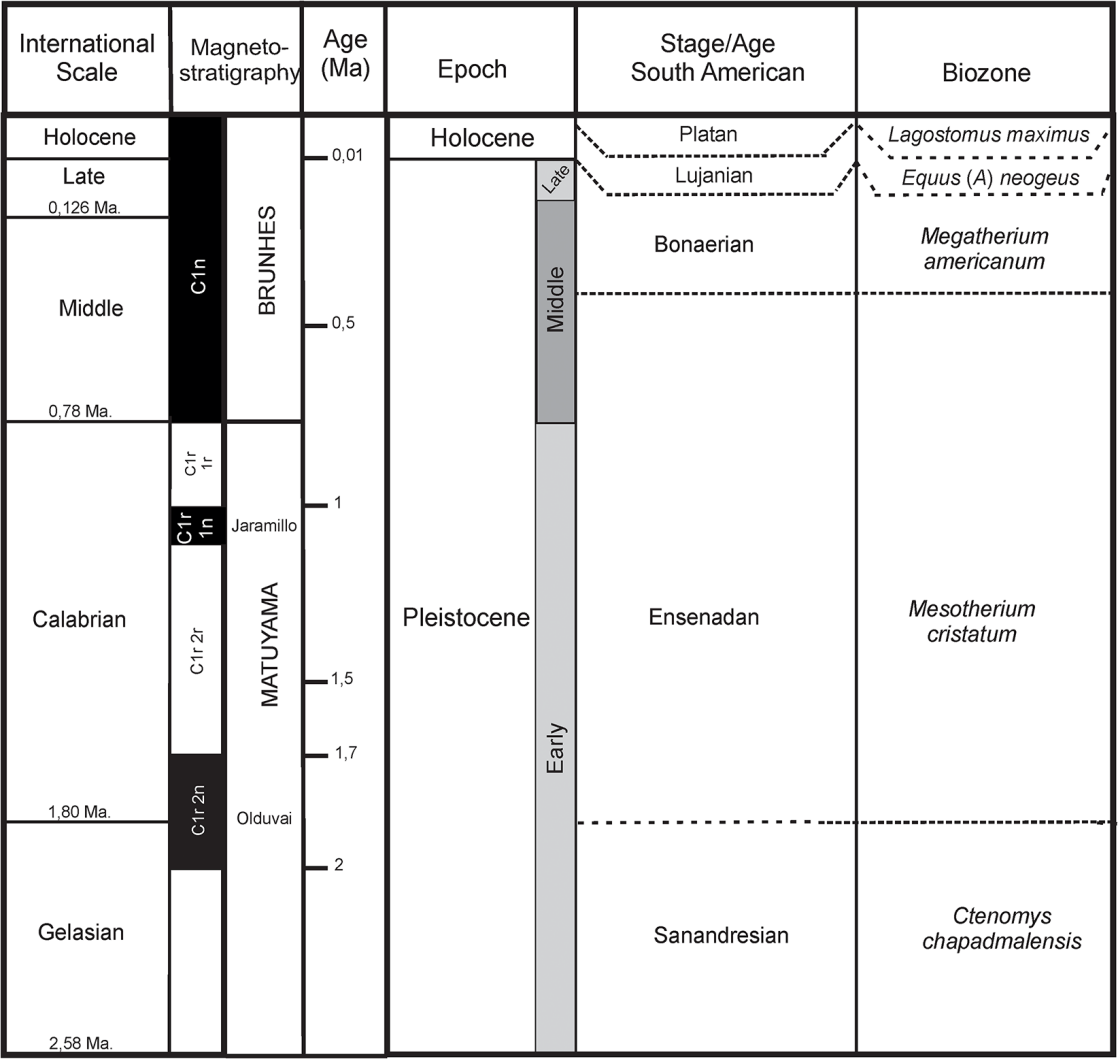
Chronological scheme with magnetostratigraphy, biozones and South American Stage/Age of the Pampean Region [42].

The type material comes from Ensenada city located in the vicinity of La Plata city, capital of Buenos Aires Province (Fig 1). La Plata has played a very important role in the study of the Argentine Quaternary because of the numerous civil excavations that rescued valuable fossil specimens, which has attracted the interest of researchers since the end of the 19th Century. Currently, the Ensenadan Stage/Age (lower to middle Pleistocene) corresponds to a chronostratigraphic unit defined for the Pampean Region (Argentina) and is based on the Biozone of *Mesotherium cristatum* [42, 45] (Fig 2). The base of the Ensenadan is currently unknown, although it is tentatively placed around 2 Ma BP and its upper limit is close to 0.40 Ma BP [45, 46] and extends from the lower part of Chron Brunhes (ca. 0.78 Ma) to more than 0.98 Ma (subhcron C1r1n). Thus defined, the Ensenadan covers a wide time span probably surpassing 1.6 Ma, where numerous environmental changes that affected the faunal composition have been recorded [47, 48, 45, 49]. The assignment of the levels with *T*. *mirus*, MLP 54-III-16-1(División Paleontología Vertebrados, Facultad de Ciencias Naturales y Museo, Universidad Nacional de La Plata, La Plata, Argentina) to the Ensenadan Stage/Age is justified by the presence of *M*. *cristatum*; the fossil that defines the biozone.

The paratype (MLP 34-IV-12-6) of the new taxon was extracted in an excavation during the building of the bridge piers for the Santa Fe railroad on the Salado River (Santa Fe Province, in schedula) (Fig 1). This locality is referred to the Lujanian Stage/Age (upper Pleistocene–early Holocene) [47, 42] as *Equus* (*Amerhippus*) *neogaeus* Lund, 1840 (MLP 34-IV-12-1) was found there; the exclusive taxon that is the base for the recognition of the Lujanian Stage/Age [47].

## Materials and Methods

### Nomenclature Acts

The electronic edition of this article conforms to the requirements of the amended International Code of Zoological Nomenclature (ICZN), and hence the new names contained herein are available under that Code from the electronic edition of this article. This published work and the nomenclatural acts it contains have been registered in ZooBank, the online registration system for the ICZN. The ZooBank LSIDs (Life Science Identifiers) can be resolved and the associated information viewed through any standard web browser by appending the LSID to the prefix “http://zoobank.org/”. The LSID for this publication is**:** urn:lsid:zoobank.org:pub:6CCF91E1-866F-4CAC-B1A2-0CDEB60DA533. The electronic edition of this work was published in a journal with an ISSN (1932–6203), and has been archived and is available from the following digital repositories: PubMed Central (http://www.ncbi.nlm.nih.gov/pmc), LOCKSS (http://www.lockss.org).

Comparisons were made with homologous materials from Pliocene and Pleistocene species of *Pampatherium*: *P*. *humboldtii* (Lund, 1839) *P*. *typum* (Gervais and Ameghino, 1880) and *P*. *mexicanum* Edmund, 1996; and *Holmesina*: *H*. *floridana* (Robertson, 1976), *H*. *septentrionalis* (Simpson, 1930), *H*. *occidentalis* (Hoffstetter, 1952), *H*. *paulacoutoi* (Cartelle and Bohórquez, 1985) and *H*. *rondoniensis* Góis, Scillato-Yané, Carlini and Ubilla, 2012; and also with the related genera *Scirrotherium*: *S*. *hondaense S*. *antelucanus* Laurito and valerio, 2013, and *S*. *carinatum*; *Vassallia*: *V*. *minuta* (Moreno & Mercerat, 1891) *Kraglievichia*: *K*. *paranensis* and *Plaina*: *P*. *intermedia* (Ameghino, 1888) Comparisons were also made with Dasypodidae: *D*. *puntactus* Lund, 1840 and Glyptodontidae: *Propalaehoplophorus australis* Ameghino, 1887 and *Neosclerocalyptus ornatus* (Owen, 1845) (see Appendix).

**Fig 3:**
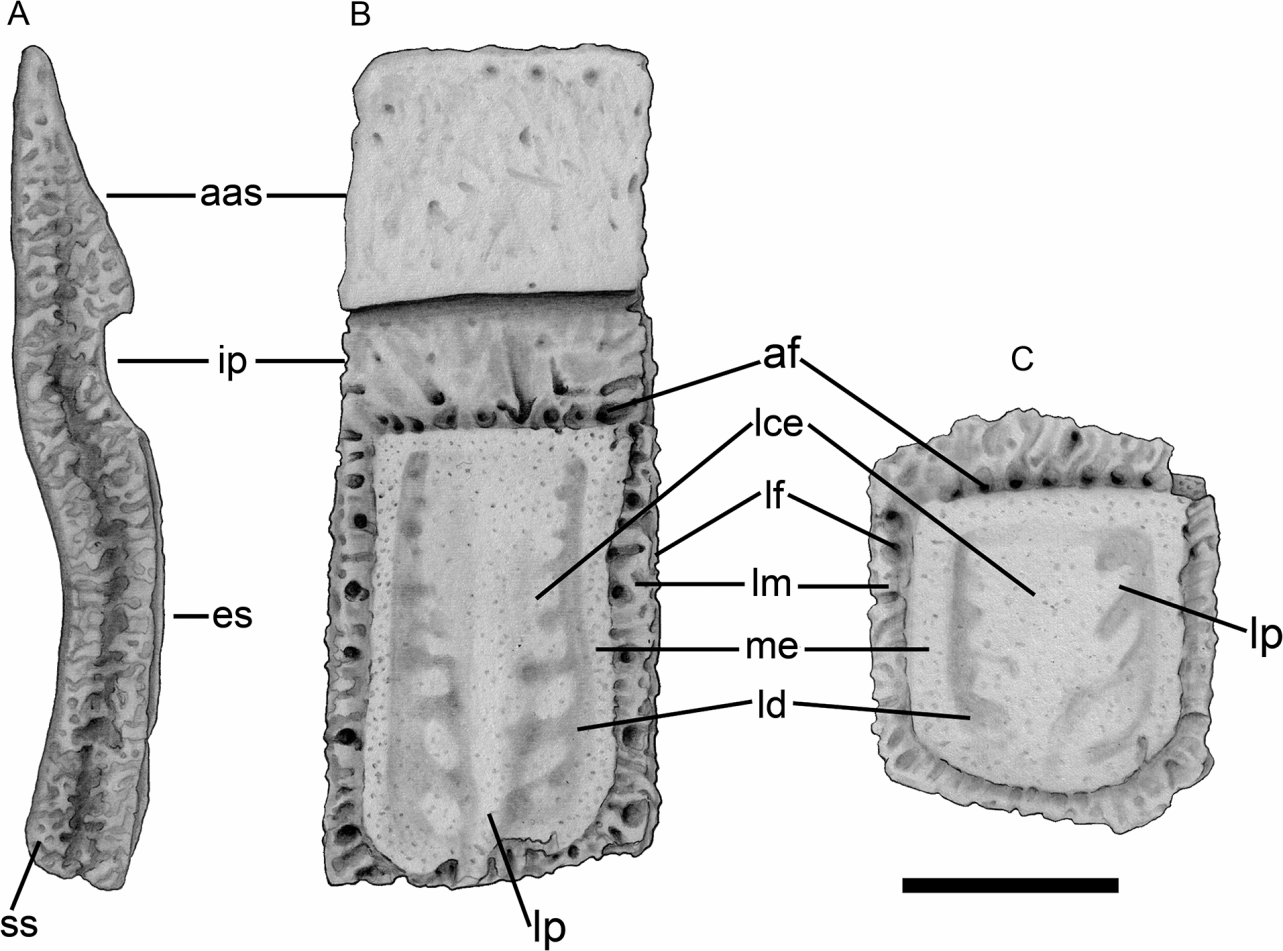
Terminology for Pampatheriidae osteoderms [31]. **A–B**, semi-movable osteoderm of the *Tonnicinctus mirus* gen. et sp. nov.; **C,** fixed osteoderm of the scapular buckler, illustrations of the *Tonnicinctus mirus* gen. et sp. nov. (holotype, MLP 54-III-16-1). Abbreviations: **aas**, anterior articular surface; **af**, anterior foramina; **es**, exposed surface; **id**, intermediate portion; **lce**, longitudinal central elevation; **ld**, longitudinal depressions; **lf**, lateral foramina; **lm**, lateral margins; **me**, marginal elevation; **lp**, lateral projection; **ss**, sutural surface. Scale bars = 50 mm.

The nomenclature used for the description of the osteoderms corresponds to that proposed by [31] for Pampatheriidae osteoderms (Fig 3). Some authors consider that the recognition of new taxa based exclusively on the characters of the osteoderms can, sometimes, overestimate the taxonomic diversity [50, 23, 51]. However, when the evaluation of the osteoderms from homologous regions is possible, the ornamental pattern is a valid criterion for the recognition of extant and extinct species [40, 52, 53, 31].

The morphological characters described by [21, 54, 55, 56, 57, 58, 59, 24, 26, 30, 32] were used for the cranial and postcranial elements of the holotype. All measurements were taken with a mechanical caliper (precision of 0.5 mm).

## Results

### Systematic Paleontology

Xenarthra Cope, 1889

Cingulata Illiger, 1811

Glyptodontoidea Gray, 1869

Pampatheriidae Paula Couto, 1954

*Tonnicinctus* gen. nov. urn:lsid:zoobank.org:act:F1662DFD-693B-461A-9D5A-93041767D582.

**Type species**—*Tonnicinctus mirus* gen. et sp. nov. urn:lsid:zoobank.org:act:F1662DFD-693B-461A-9D5A-93041767D582.

**Etimology**—“*Tonni*”, in tribute to Dr. Eduardo P. Tonni, a distinguis hed Argentine paleontologist. He has significantly contributed through his studies to the knowledge of fossil birds and mammals and to the Argentine geology and Quaternary biostratigraphy. From the Latin “*cinctus*”, it means to the mobile bands present in the caparace of Pampatheriidae. The specific epithet "*mirus*" means surprising/wonderful, due to the asymmetric and unique drawing present in each osteoderm.

**Holotype**—MLP 54-III-16-1, 27 osteoderms from the dorsal caparace: five fixed (complete) and five semi-movable (incomplete) from the scapular buckler, one fixed (complete) and two semi-movable (complete) from the pelvic buckler, four movable (incomplete) from the movable bands, and ten articular regions from the movable bands or semi-movable from the scapular buckler; right temporal region of the skull; and postcranium represented by two thoracic vertebrae, right femur with the head, third trochanter and patellar crest partially broken and right patella.

**Type Locality and Age**—Colón street, Ensenada city (34° 51’ S y 57° 54W), column 32, perforation number 140, 17 meters deep (Buenos Aires Province). Ensenadan Stage/Age (lower–middle Pleistocene).

**Paratype**—MLP 34-IV-12-6, one movable or semi-movable osteoderm (broken), from the pelvic buckler.

**Type Locality and Age of the Paratype**—Salado River (31° 38’ S and 60° 42’W) (Santa Fe Province). Age: Lujanian Stage/Age (upper Pleistocene–early Holocene). (Frenguelli Collection, in schedula).

**Diagnosis**—Medium size, larger than *Plaina intermedia* but smaller than *Pampatherium* and *Holmesina*. Osteoderms have a greater denticulated sutural surface than *Pampatherium typum* but less than *Holmesina paulacoutoi*. Like *Holmesina*, fixed osteoderms have wide and very rugged anterior and lateral margins, wider and higher marginal elevation in all the perimeter than *S*. *antelucanus*, *Vassallia minuta*, *Plaina* and *Pampatherium*, longitudinal depressions less shallow than *Scirrotherium carinatum*, *Kraglievichia paranensis* and *Holmesina*, and a very different longitudinal central elevation from any Pampatheriidae: lower than the marginal elevation, wide, asymmetric and with small and irregular lateral projections. As in *Pampatherium*, movable osteoderms develop a high and rugged intermediate portion, wide lateral margins with large, deep and separated foramina, but the marginal elevation is wider and higher than in *Pampatherium*; longitudinal depressions are narrower and shallower than in *Holmesina*. The longitudinal central elevation is wide, anteriorly depressed, confluent with the marginal elevation and reaching the posterior margin, more asymmetrical than in the fixed osteoderms, and has grossly oval irregular lateral projections. Semi-movable osteoderms of the pelvic buckler similar to the movable osteoderms, but with anterior and laterals wider margins, narrow and deep longitudinal depressions, much wider and asymmetric longitudinal central elevation, and more irregular lateral projections. Femur larger than *Kraglievichia* (i.e., cf. *K*. *paranensis*) and *Holmesina floridana*, but smaller than *Pampatherium humboldtii* and *H*. *paulacoutoi*; Greater trochanter shorter than *P*. *humboldtii* and *Kraglievichia* (i.e., cf. *K*. *paranensis*) but longer than *H*. *paulacoutoi*; Third trochanter greater and more distanced from the head than *Kraglievichia* (i.e., cf. *K*. *paranensis*) and *H*. *floridana*, and wider and rugged than any other Pampatheriidae; contrary to *Kraglievichia* (i.e., cf. *K*. *paranensis*), *H*. *floridana* and *P*. *humboldtii* the lesser trochanter is longer than wider. Trochanteric fossa wider and deeper than *Kraglievichia* (i.e., cf. *K*. *paranensis*) and *H*. *paulacoutoi*.

## Comparative Description

### Caparace

**Fig 4:**
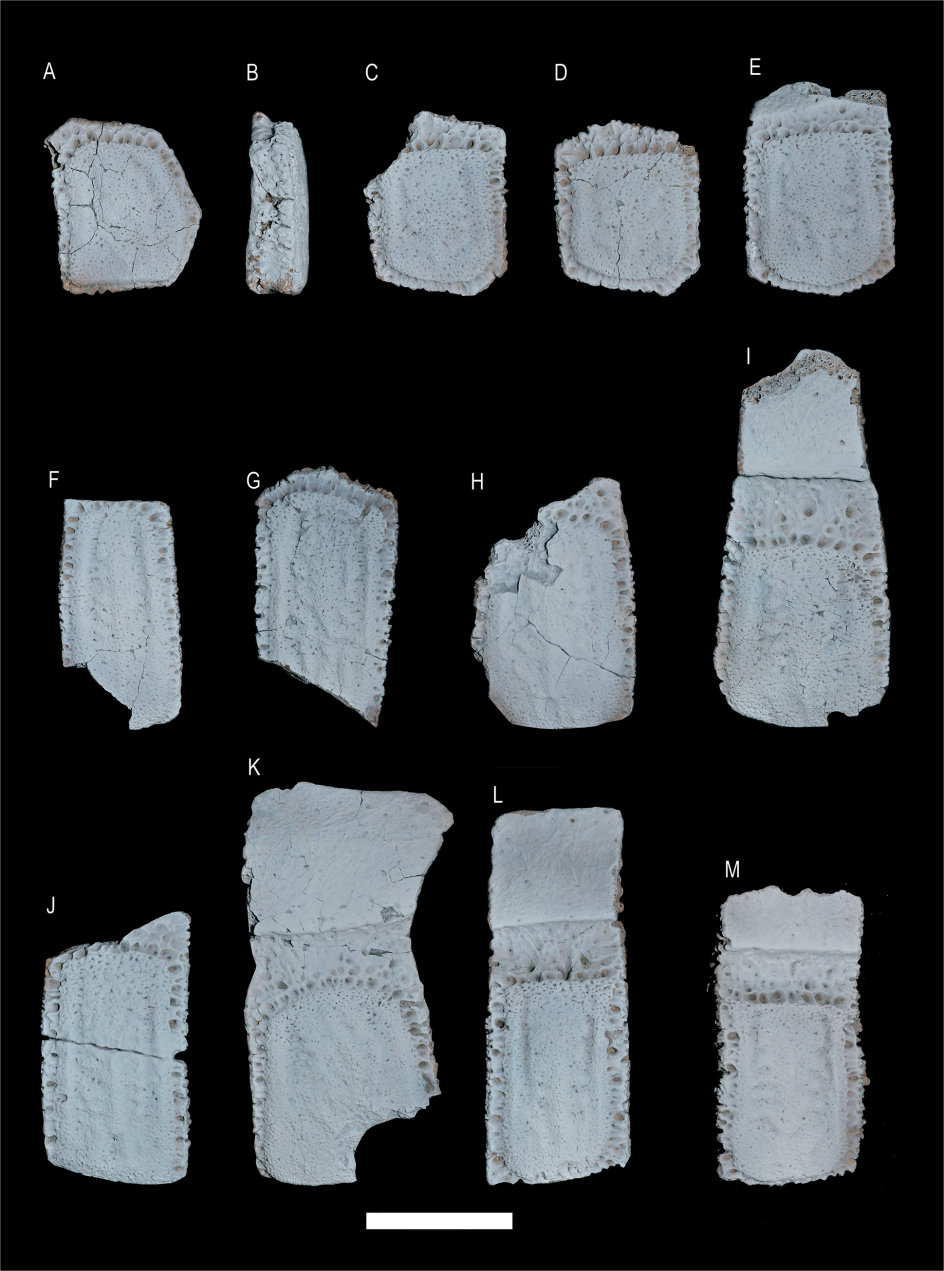
*Tonnicinctus mirus* gen. et sp. nov. (holotype, MLP 54-III-16-1). Osteoderms from different regions of the carapace. **A–D**, fixed osteoderms of the scapular buckler; **E**, fixed osteoderm of the pelvic buckler; **F–G**, semi-movable osteoderms of the last row of the scapular buckler; **H–K**, movable osteoderms; **L–M**, semi-movable osteoderms of the first row of the pelvic buckler. Scale bars = 30 mm.

**Fig 5:**
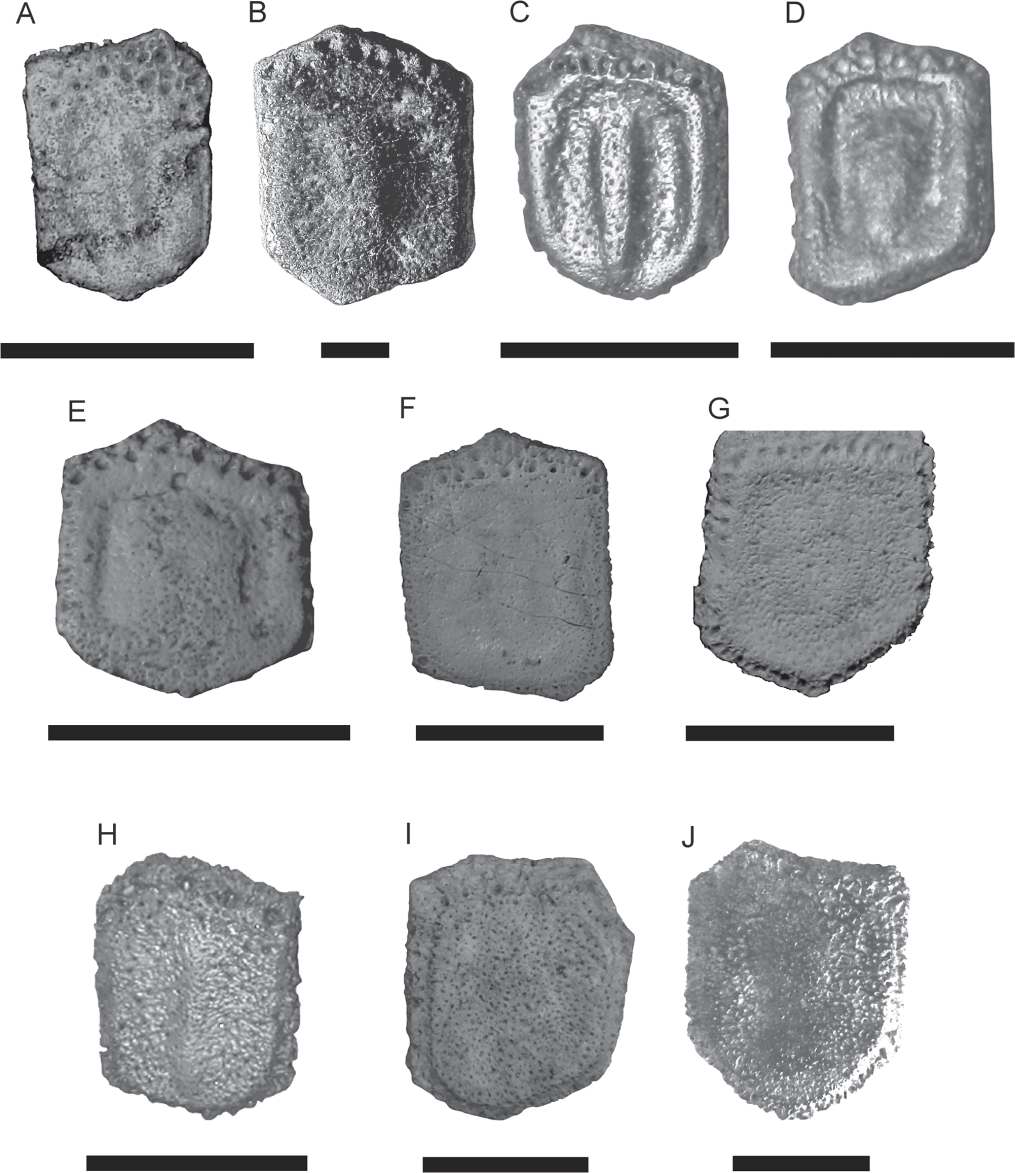
Comparison of fixed osteoderms. **A,** *Scirrotherium hondaense* (UCMP 37979), scale bars = 50 mm; **B,** *S*. *antelucanus* (CFM-2559), scale bars = 10 mm; **C,** *S*. *carinatum* (MLP 41-XII-13-905), scale bars = 30 mm; **D,** *Kraglievichia paranensis* (MLP 69-IX-8-13), scale bars = 30 mm; **E,** *Vassallia minuta* (MLP 69-XII-26-17), scale bars = 30 mm; **F,** *Plaina intermedia* (FMNH P 14424), scale bars = 30 mm; **G,** *Pampatherium humboldtii* (MLP 81-X-30-1), scale bars = 30 mm; **H,** *Holmesina floridanus* (UF 224397), scale bars = 30 mm; I**,** *H*. *septentrionalis* (AMNH 23435), scale bars = 30 mm; **J,** *H*. *paulacouoti* (holotype, MCL-501/110-126), scale bars = 30 mm.

**Fixed osteoderms from the scapular and pelvic buckler**—these osteoderms possess a generally pentagonal or hexagonal shape in the scapular buckler and a quadrangular shape in the pelvic buckler (Figs 3C, 4A–4E and 5A–5J). In *Tonnicinctus mirus*, the anterior and lateral margins are very wide and rugged, more than in *Pampatherium typum*, *P*. *humboldtii*, MCL 900 (Museu de Ciências Biológicas da Pontifícia Universidade Católica de Minas Gerais, Belo Horizonte, Brazil); *P*. *mexicanum*, INAH 6201 (Instituto Nacional de Antropología e Historia, Ciudad de México, Distrito Federal, México) but less than in *Holmesina paulacoutoi* (Fig 5G and 5J). The foramina of the anterior margin are much larger than those of the lateral margins, and are larger and deeper than in *Pampatherium* and smaller than in *H*. *paulacoutoi* (Table 1).

**Table 1:** Comparison of fixed osteoderms from the buckler of *Tonnicinctus mirus* gen. et sp. nov. (holotype, MLP 54-III-16-1) with other pampatheres.

Taxon	Osteoderm type	Length average	Width average	Mean area average
*T*. *mirus*	Scapular	39.8	30.7	1221.86
	Pelvic	48.5*	34.2*	1316.7*
*S*. *hondaense*	Scapular	19.75	19.5	385.12
	Pelvic	22.8	18.66	425.44
*S*. *carinatum*	Scapular	30*	26.1*	783*
	Scapular	36.16	27.71	1001.99
*S*. *antelucanus*	Scapular	26.9	20.8	559.52
*V*. *minuta*	Pelvic	31	23.75	736.25
	Scapular	34.8	31.9	1110.12
*Pl*. *intermedia*	Scapular	44*	34*	1496*
*Pl*. *brocherense*	Scapular	37.3	26.7	995.91
*K*. *paranensis*	Scapular	46	37*	1702
*H*. *septentrionalis*	Scapular	49.6	41	2033.6
*H*. *major*	Scapular	62.9	52.5	3286.5
	Scapular	56.5	37.8	2135.7
*H*. *paulacoutoi*	Pelvic	63	51.5	3244.5
*P*. *mexicanum*	Scapular	40*	32*	1280*
*P*. *typum*	Pelvic	33.4	30.3	1012.02
*P*. *humboldtii*	Scapular	45*	31*	1395*

*Not average (only one osteoderm).

**When there is only one type of osteoderm is because the other is unknown.

The marginal elevation is wider and its contour is visible in all around the osteoderm perimeter than in *Scirrotherium hondaense*, UCMP 37979 (University of California Museum of Paleontology, California, USA); *S*. *antelucanus*, CFM-2559 (Colección de Fósiles y Minerales del Departamento de Historia Natural del Museo Nacional de Costa Rica) and *Vassallia minuta* (Fig 5A, 5B and 5E) and *Pampatherium*, but less marked than in *S*. *carinatum*, *K*. *paranensis* and *Holmesina* (Fig 5C, 5H and 5J). The longitudinal depressions are much deeper than in *Pl*. *intermedia*, FMNH P 14424 (Field Museum Natural History, Chicago, USA) and *Pampatherium* but less deeper than in *S*. *carinatum*, *K*. *paranensis* and *Holmesina*. The longitudinal central elevation is different from any other Pampatheriidae, and it is noticeable for its particular morphology in each osteoderm that presents some asymmetry but always in a pattern. This elevation is confluent with the anterior margin, proximally wide and with rounded and very asymmetric lateral projections in both sides; in contrasts, in other Pampatheriidae, like *S*. *carinatum* (Fig 5C), the elevation have a very marked ridge or is be very diffuse like in *P*. *intermedia* (Fig 5F).

**Semi-movable osteoderm from the scapular buckler**—unlike movable osteoderms, they do not possess a movable anterior articular surface (Figs 3A and 3B, 4F and 4G). The only preserved osteoderm of this category has its distal portion broken, although the decrease in the thickness along the preserved osteoderm indicates a beveled posterior margin. The anterior and lateral margins are wider than in *K*. *paranensis*, *Scirrotherium*, *Pl*. *intermedia* and *Pampatherium*, but narrower than in *Holmesina*. This osteoderm has a very rough texture and the anterior foramina are more abundant, smaller and more irregularly distributed than the osteoderms from the scapular buckler.

The marginal elevation is much thinner and higher than in the fixed osteoderms of the scapular buckler and it is more sculpted than in *Pampatherium* but less sculpted than in *K*. *paranensis*. The longitudinal depressions are narrower and deeper than in *V*. *minuta*, *P*. *intermedia* and *P*. *typum*, but wider than in *K*. *paranensis*, *S*. *antelucanus*, *S*. *carinatum* and *Holmesina*. The longitudinal central elevation is not straight throughout all its length like in other Pampatheriidae, but this elevation curves externally at the half of the osteoderm and the lateral projections are more numerous, asymmetrical and higher (Table 2).

**Table 2:** Comparison of semi-movable and movable osteoderms of *Tonnicinctus mirus* gen. et sp. nov. (holotype, MLP 54-III-16-1) with other pampatheres.

Taxon	Osteoderm type	Length average	Width average	Mean area average
*T*. *mirus*	Semi-movable	76.25	32.7	2493
*S*. *hondaense*	Movable	60*	25*	1500*
*S*. *carinatum*	Semi-movable	42.21	24.31	1026
*V*. *minuta*	Semi-movable	59.9	24.9	1491
	Semi-movable	66	28	1848
*K*. *paranensis*	Movable	60.5*	26.5*	1603*
	Semi-movable	58.25	31.5	1834
*Pl*. *intermedia*	Movable	53*	32.5*	1722*
*Pl*. *brocherense*	Movable	58**	39**	2262**
*H*. *paulacoutoi*	Movable	83*	36*	2988*

*Not average (only one osteoderm).

**When there is only one type of osteoderm is because the other is unknown.

**Fig 6:**
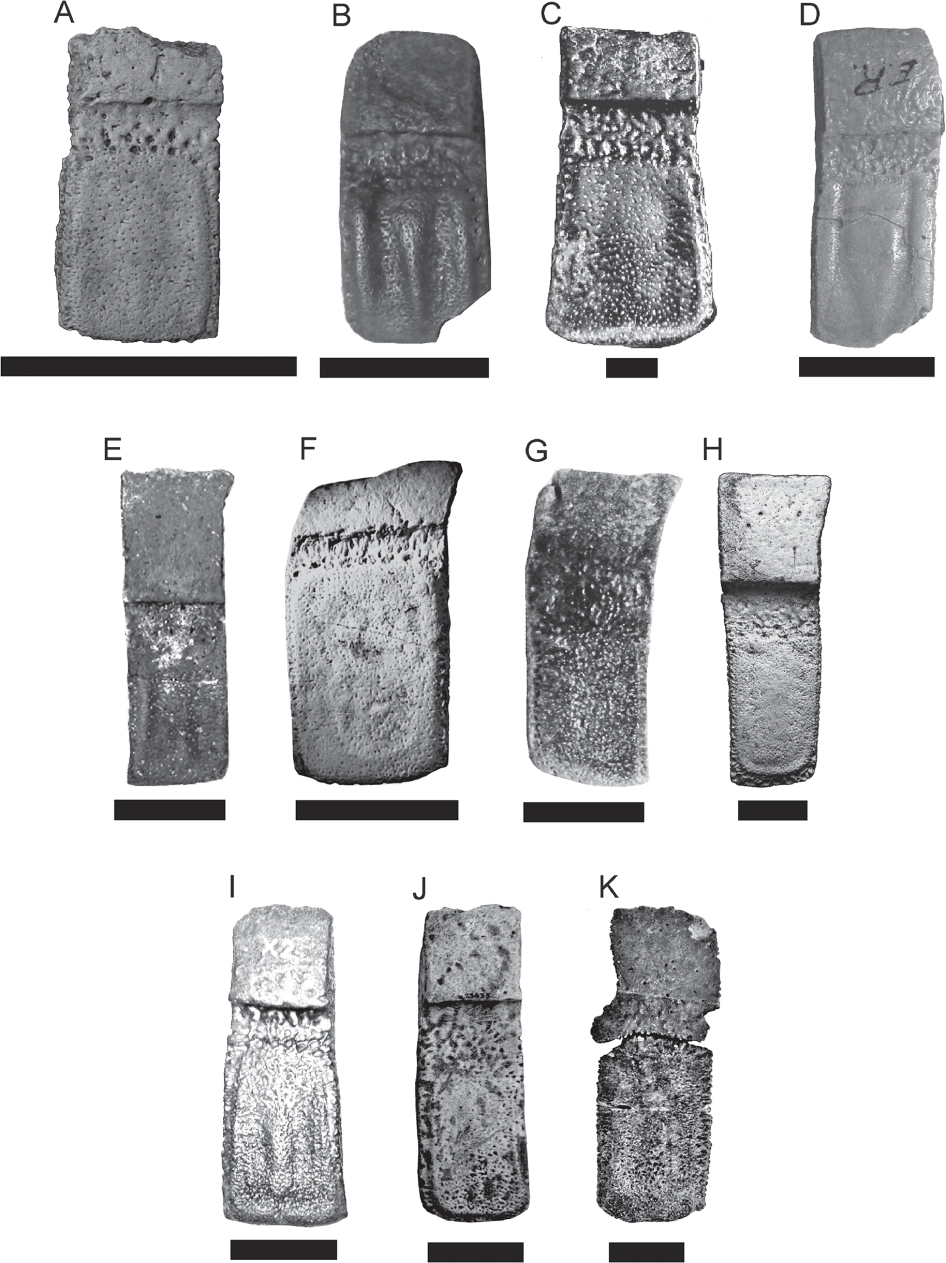
Comparasion of movable osteoderms. **A,** *Scirrotherium hondaense* (paratype, UCMP 40056), scale bars = 30 mm; **B,** *S*. *carinatum* (holotype, MLP 69-IX-8-13AB), scale bars = 30 mm; **C,** *S*. *antelucanus* (holotype, CFM-2867), scale bars = 10 mm; **D,** *Kraglievichia paranensis* (MLP 69-IX-8-13), scale bars = 30 mm; **E,** *Holmesina floridanus* (UF 224397), scale bars = 30 mm; **F,** *Plaina intermedia* (FMNH P 14424), scale bars = 30 mm; **G,** *Pampatherium typum* (MLP 52-IX-28-20), scale bars = 30 mm; **H,** *P*. *mexicanum* (holotype, INAH 6201), scale bars = 30 mm; **I,** *H*. *occidentalis* (ROM 28392), scale bars = 30 mm; **J,** *H*. *septentrionalis* (AMNH 23435), scale bars = 30 mm; **K,** *H*. *paulacoutoi* (holotype, MCL 501/110-126), scale bars = 30 mm.

**Osteoderms of the movable bands**—the movable band region, composed by successive imbricated osteoderms, forms the intermediate region of the caparace (Figs 4H, 4L and 6A–6K). The number of movable bands can differ greatly in living and extinct taxa (Dasypodidae), however, in all Pampatheriidae where this region is conserved (*P*. *intermedia*, *Holmesina septentrionalis*, AMNH 23435 (American Museum of Natural History, New York, USA) and *Pampatherium humboldtii*) have three movable bands in the caparace; which would be a conservative character for the family [13, 31, 30]. The movable (= imbricated) osteoderms have a bigger rough intermediate portion than in *Scirrotherium*, *K*. *paranensis*, *V*. *minuta*, *P*. *intermedia*, *Holmesina* but smaller than in *Pampatherium* (Fig 6A–6K). Like in *H*. *paulacoutoi*, the lateral margins are wider and with the foramina very separated. The foramina of the intermediate portion are numerous and large, generally arranged in a main line that is distally convex (Table 2).

The marginal elevation is wider than in the semi-movable osteoderms of the pelvic buckler. The longitudinal depressions are narrower and shallower than in *S*. *carinatum*, *K*. *paranensis*, *H*. *occidentalis*, ROM 28392 (Royal Ontario Museum, Toronto, Canada) and *H*. *septentrionalis*, but deeper than in *Pl*. *intermedia* and *P*. *typum*. The longitudinal central elevation in movable osteoderms and semi-movable osteoderms from the scapular buckler is more complex than in fixed osteoderms. The elevation is wide proximally and in some osteoderms there is no distinction between longitudinal central elevation and marginal elevation. The lateral projections are very irregular and some of them emerge from the central elevation.

**Semi-movable osteoderms from the pelvic buckler**—the semi-movable osteoderms are similar to those movable ones described above (Figs 3A, 3B, 4L and 4M). Both osteoderms have an anterior articular surface and an intermediate portion, however the semi-movable osteoderms have generally a shorter intermediate portion, a constant thickness and the posterior margin is not beveled (Table 2).

The lateral margins are wider and more rugged than in the movable osteoderms and with several lateral foramina. The anterior foramina of the intermediate portion are arranged in a straight deep row. The marginal elevation is narrower and higher than in the movable osteoderms, but wider and higher than in *P*. *humboldtii* and *P*. *mexicanum* and lower than in *S*. *antelucanus*, *S*. *carinatum*, *K*. *paranensis* and *H*. *paulacoutoi*. The longitudinal depressions are narrower and deeper than in *P*. *humboldtii* and *P*. *mexicanum*, but shallower than in *S*. *carinatum*, *K*. *paranensis* and *H*. *occidentalis*. The longitudinal central elevation is much more complex than in movable osteoderms, with a more pronounced asymmetry and a higher number of lateral projections.

### Skull

**Fig 7:**
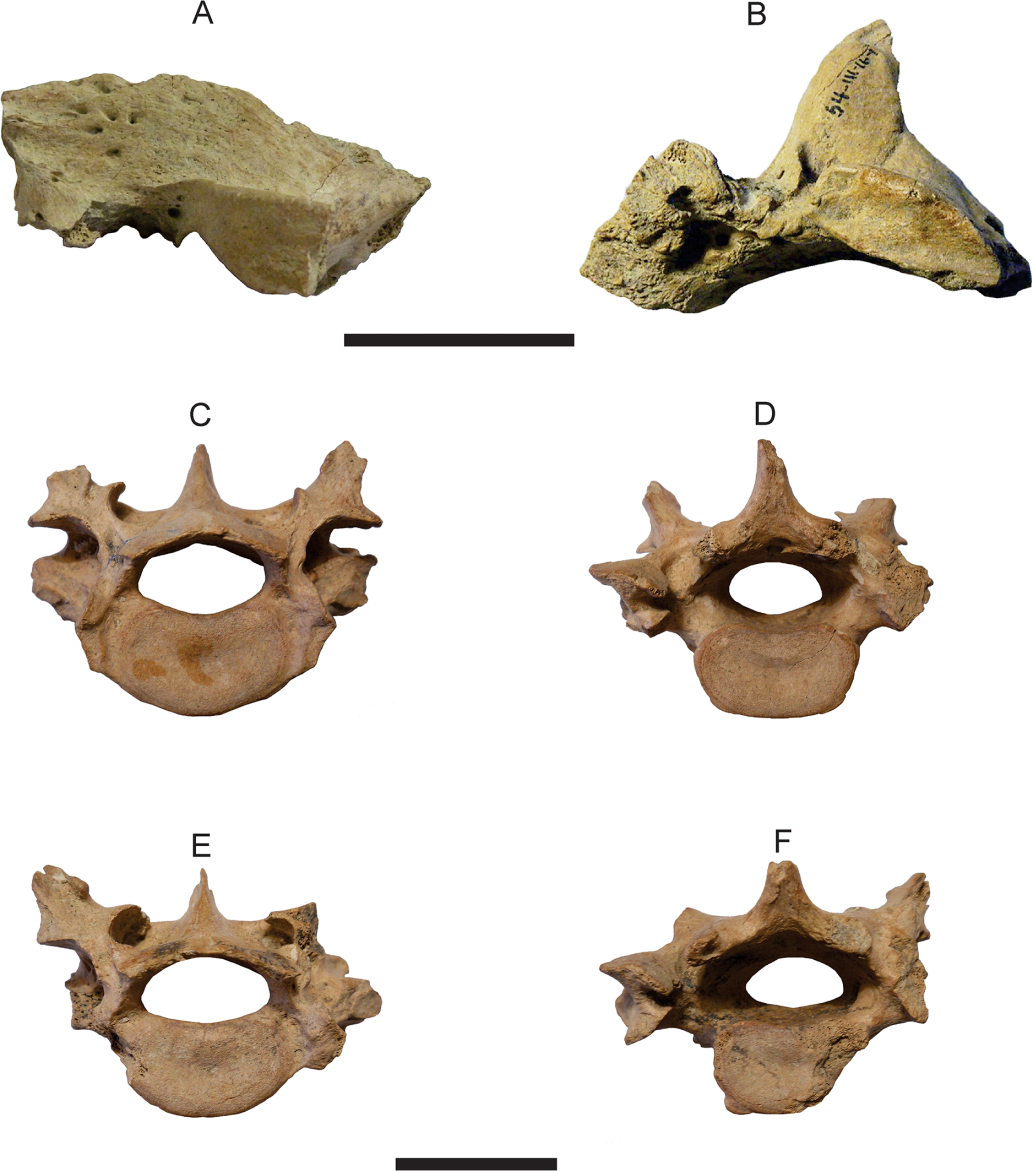
Cranial and postcranial elements of *Tonnicinctus mirus* gen. et sp. nov (holotype, MLP 54-III-16-1). **A–B,** lateral and ventral view of the temporal region; **C–E,** anterior view of the thoracic vertebrae; **D–F,** posterior view of the thoracic vertebrae. Scale bars = 50 mm.

**Fig 8:**
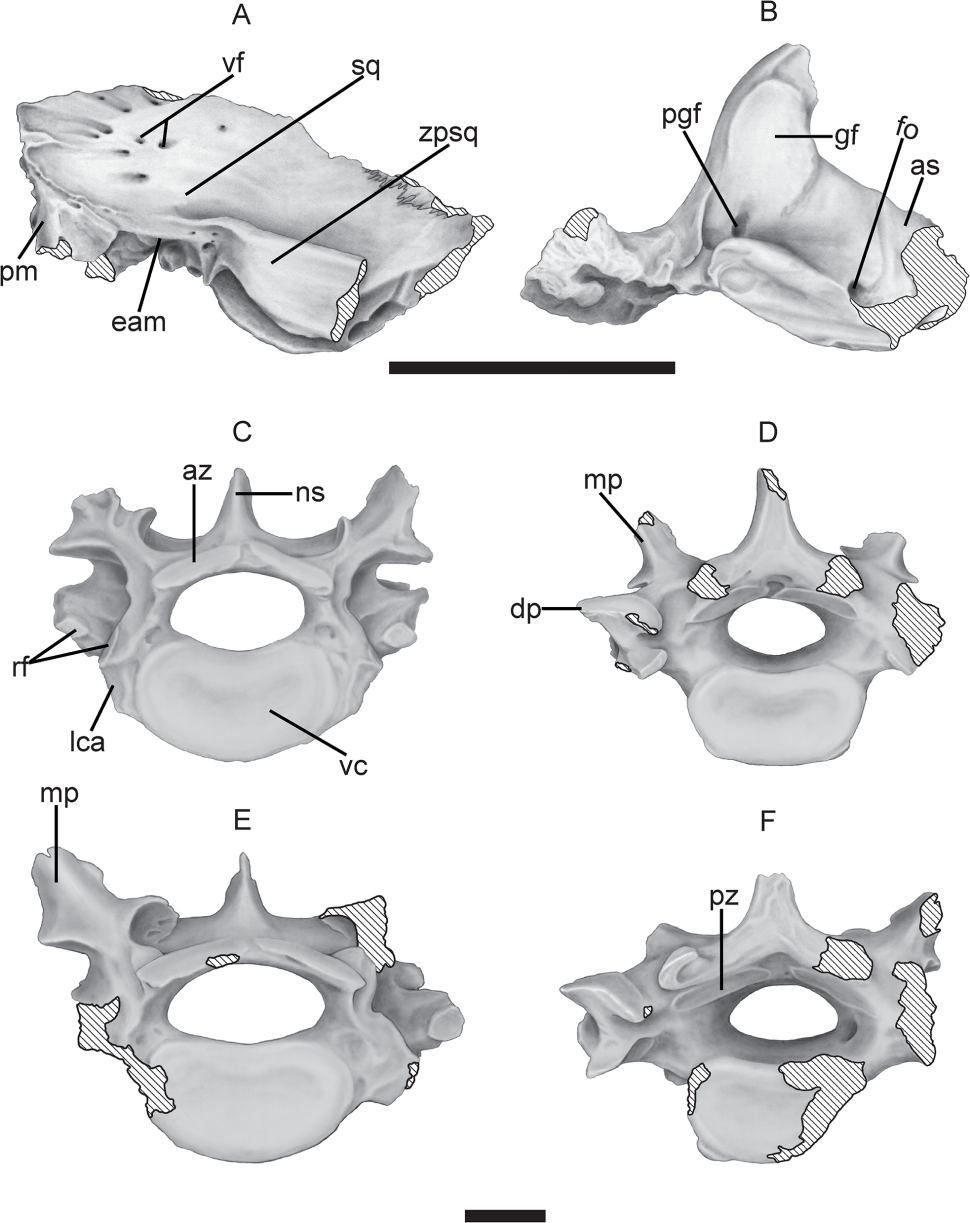
Cranial and postcranial elements of *Tonnicinctus mirus* gen. et sp. nov (holotype, MLP 54-III-16-1). Drawing of the anatomical structures mentioned in the text. **A–B,** temporal region lateral and ventral views, scale bars = 50 mm. **D–F,** thoracic vertebrae posterior views, scale bars = 20 mm. **Abbreviations: as,** alisphenoid; **az,** anterior zygapophyseal facet; **dp,** diapophysis; **eam,** external auditory meatus; **fo,** foramen ovale; **gf,** glenoid fossa; **lca,** lateral centrum articulation; **mp,** metapophysis; **pgf,** postglenoid foramen; **pm,** pars mastoidea; **pz,** posterior zygapophyseal facet; **rf,** rib facet**; sp,** spinous process **sq,** squamosal; **vc,** vertebral centrum; **vf,** vascular formanina; **zpsq,** zygomatic process of the squamosal.

**Temporal region**—the only cranial element preserved of *T*. *mirus* is the temporal region that in lateral view shows the squamosal, external auditory meatus, postglenoid process, zygomatic process of the squamosal, while in ventral view shows the glenoid fossa, postglenoid process, foramen ovale and small portion of the alisphenoid (Figs 7A, 7B, 8A and 8B). In *T*. *mirus*, the temporal region of the squamosal has a few foramina and very small vascular canals like in *H*. *rondoniensis*, MERO-P-002 (Museu do Estado de Rondônia, Porto Velho, Rondônia, Brazil) contrary to *P*. *intermedia* (FMNH 14424), this specimen was assigned to *P*. *subintermedia* by [56], but later the designation changed to *V*. *maxima* [58, 29], *P*. *typum*, *P*. *humboldtii* and *H*. *paulacoutoi*, species with more foramina and very deep and long vascular canals in the temporal region. The zygomatic root in *T*. *mirus* is parallel to the lateral margin of the squamosal, similar to *H*. *rondoniensis* and *P*. *typum*, and is inclined in *H*. *paulacoutoi*, *H*. *septentrionalis* and *H*. *floridana*, UF 191448 (Florida Museum of Natural History, Gainesville, Florida, USA). In *T*. *mirus*, the pars mastoidea preserves only a small part (Fig 7A). This structure in the pampatheres (e.g. *P*. *intermedia*, FMNH P 14424; *P*. *typum*, MACN 11543 (Museo Argentino de Ciencias Naturales “Bernardino Rivadavia”, Buenos Aires, Argentina) has a large and robust (short and wide in *Euphractus sexcinctus* Linnaeus, 1758) occipital exposure and forms the ventrolateral corner of the occiput [56]. The external auditory meatus in pampatheres is located laterally forward and was farther posterior to the glenoid fossa that in other Dasypodidae, a similar feature with the glyptodonts [56, 29] (Fig 7A).

In ventral view, in the zygomatic process of the squamosal the glenoid fossa is located (this structure is called the glenoid cavity by [54], whereas it is called glenoid surface by [56] (Fig 8B). The fossa differs from that of other Dasypodidae and strongly resembles the fossa of the glyptodonts (56, 29). There are no remarkable differences in the glenoid fossa with others pampatheres.

### Postcranial skeleton

**Fig 9:**
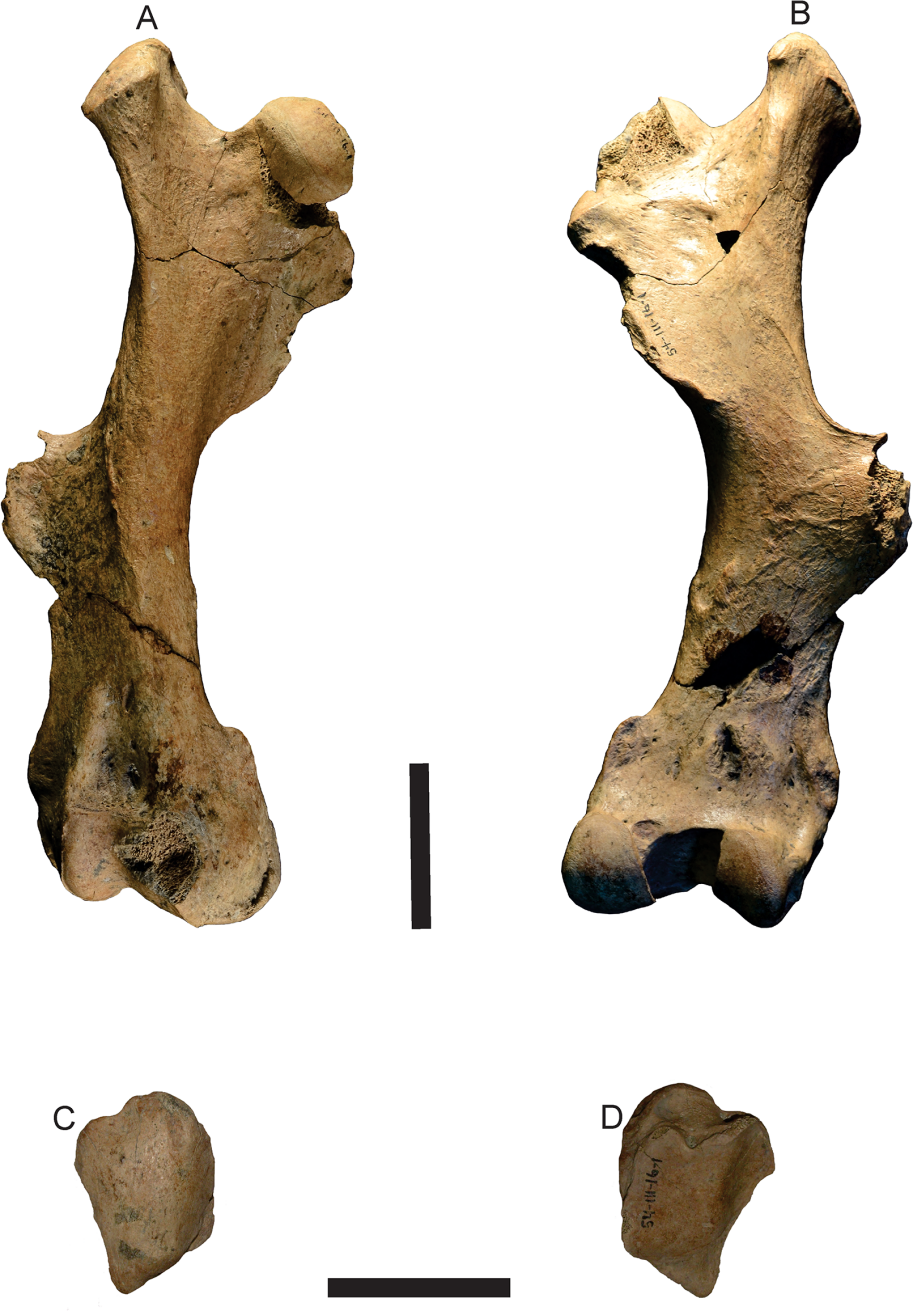
Postcranial elements of *Tonnicinctus mirus* gen. et sp. nov. (MLP 54-III-16-1). **A–B,** anterior and posterior view of the right femur; **C–D,** anterior and posterior view of the right patella. Scale bars = 50 mm.

**Thoracic vertebrae**—two posterior thoracic vertebrae are preserved (Figs 7C–7F and 8C–8F). These vertebrae belong to the typical xenarthral type, common to most Xenarthra (xenarthrous articulations, see [59]). There are two additional pairs of both the anterior and the posterior zygapophyses. In *T*. *mirus* the spinous process is considerably shorter than in *H*. *septentrionalis*, HMNS 173 (Houston Museum of Natural Science, Houston, Texas, USA) and ROM 3854 [55,15] (Figs 1* and 9*, respectively) and proportionally shorter that in *D*. *puntactus*, MN 552-V (Museu Nacional, Rio de Janeiro, Brazil). The angle produced by the transverse processes in pampatheres is much wider than 90° unlike in *D*. *puntactus* (with approximately a 90° angle [60] see Fig 6C* and 6D*) and the anterior zygopophyses are not as widely separated as in *D*. *puntactus*. The width and depth of the centrum is relatively smaller in pampatheres, but the length is proportionally the same as in *D*. *puntactus*. The largest and smallest diameters of the vertebral foramen are smaller in *T*. *mirus* than in *H*. *septentrionalis* (Table 3).

**Table 3:** Comparison of the last thoracic vertebrae.

	A	B
Aplc	28.5	30
Gdcp	40	40
Gdca	33	35
Gdvfp	39	40
Gdvfa	26	25
Sdvfp	20	21
Sdvfa	16	20

**A,** *T*. *mirus* gen. et sp. nov. (holotype, MLP 54-III-16-1); **B,** *H*. *septentrionalis* (HMNS 173). **Abbreviations: Aplc,** Antero-posterior length of the centrum; **Gdcp,** posterior view of the greatest diameter of the centrum; **Gdca,** Greatest diameter of centrum anterior face; **Gdvfp,** Greatest diameter of vertebral foramen posterior views; **Gdvfa,** Greatest diameter of vertebral foramen anterior views; **Sdvfp,** Smallest diameter of vertebral foramen posterior views; **Sdvfa,** Smallest diameter of vertebral foramen anterior views.

**Fig 10:**
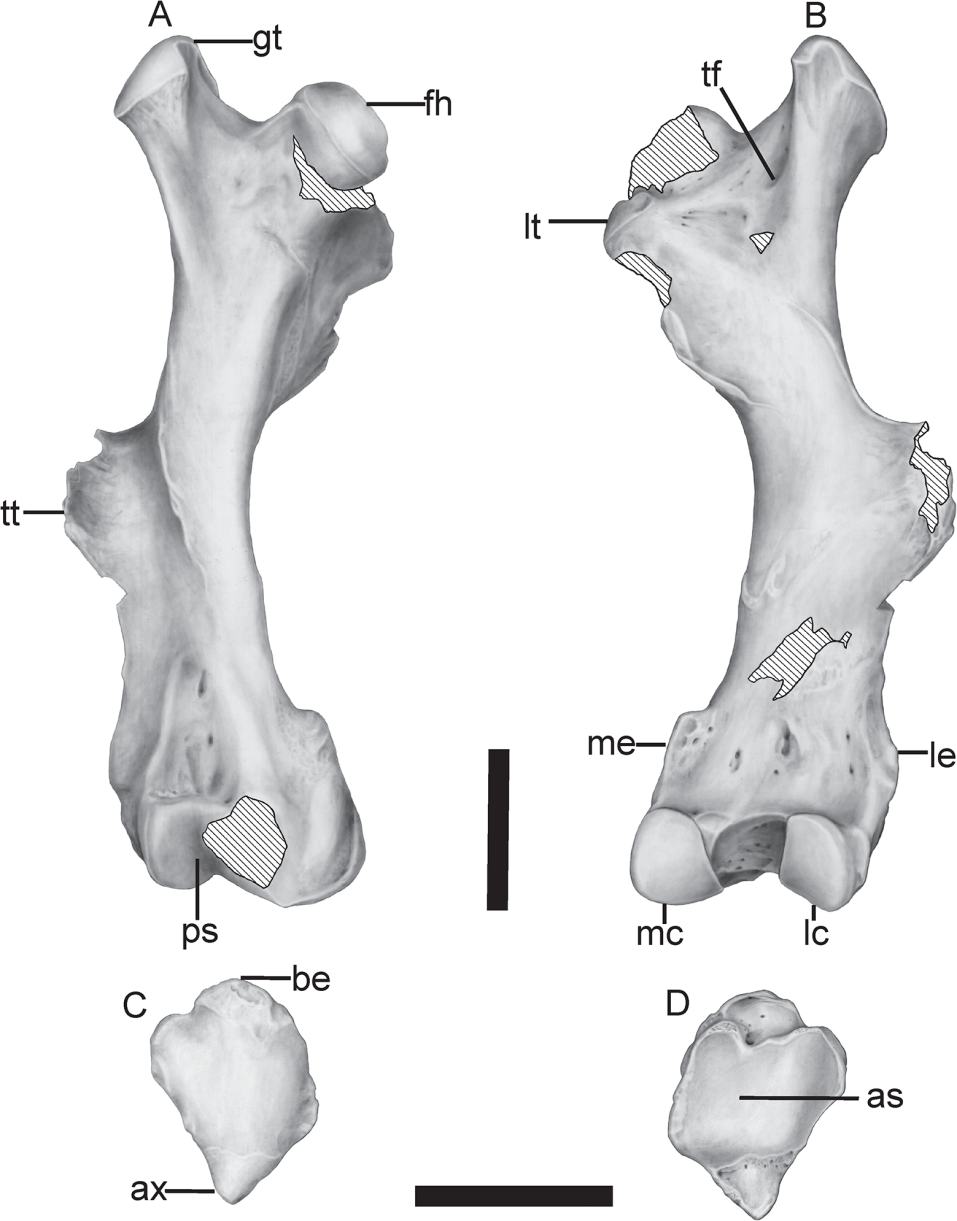
Postcranial elements of *Tonnicinctus mirus* gen. et sp. nov. (MLP 54-III-16-1). Drawing of the anatomical structures mentioned in the text. **A–B,** anterior and posterior view of the right femur; **C–D,** anterior and posterior view of the right patella. **Abbreviations: as,** articular surface; **ax,** apex; **be,** base; **fh**, femoral head; **gt,** greater trochanter; **lc,** lateral condyle; **le**, lateral epicondyle; **lt,** lesser trochanter; **mc,** medial condyle; **me,** medial epicondyle lateral; **ps,** patella surface, **tf,** trochanteric fossa; **tt,** third trochanter. Scale bars = 50 mm.

**Fig 11:**
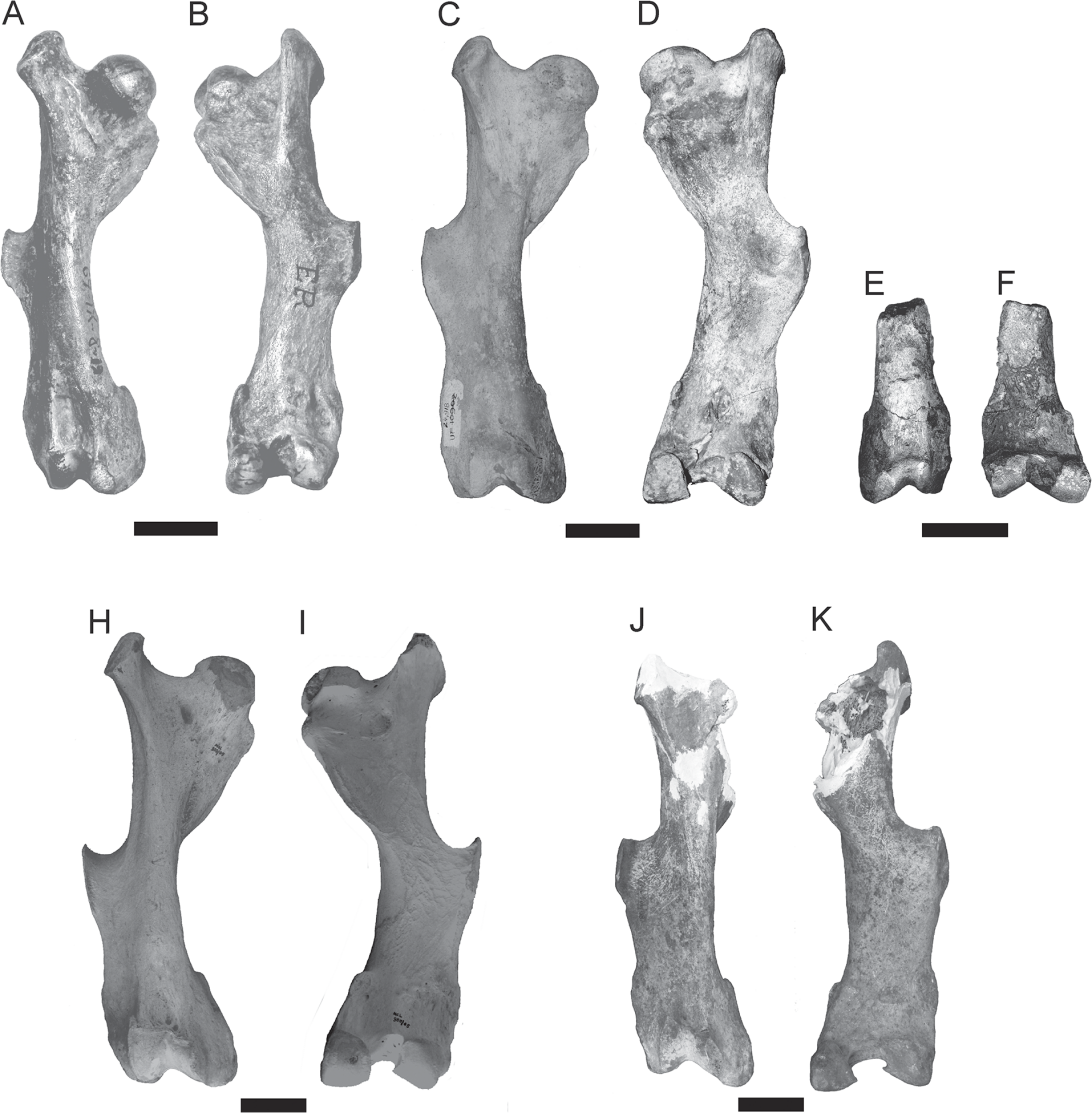
Comparison of the right femur of the other pampatheres. **A–B,** *Kraglievichia paranensis* (MLP 69-IX-8-13A) anterior and posterior view, scale bars = 30 mm; **C–D,** *Holmesina floridana* (UF 24918) anterior and posterior view, scale bars = 30 mm; **E–F,** *Scirrotherium antelucanus* **(**CFM-1639) anterior and posterior view, scale bars = 50 mm; **G–H,** *Pampatherium humboldtii* (MCL 900/05), scale bars = 50 mm; **I–J,** *H*. *paulacoutoi* (holotype, MCL 501/08), scale bars = 50 mm.

**Femur**—In *T*. *mirus*, the greater trochanter on the proximal end is highly developed, laterally compressed and with its main diameter anteroposteriorly oriented (Figs 9A, 9B, 10A, 10B and Table 4). Like in *Kraglievichia* (i.e., cf. *K*. *paranensis*) (Fig 11A and 11B) and *P*. *humboldtii* (Fig 11H and 11I), the greater trochanter exceeds in height the head of the femur, but this difference is less marked in *H*. *floridana* (Fig 11C and 11D). In contrast, in *H*. *paulacoutoi* (MCL 501/08), the height of the greater trochanter does not exceed the head of the femur, a shared feature of some glyptodonts (e.g. *N*. *ornatus* neotype, MLP 16–28).

**Table 4:** Comparison of the femur of *Tonnicinctus mirus* gen. et sp. nov. with other pampatheres.

	A	B	C	D	E	F
Gl	164	193.5	280	290	359	347
Wtt	33.5	41.3	62	70	83	72
Wde	38	51.5	70	86	91	92

**A**, *Kraglievichia* cf. *K*. *paranensis* (MLP 69-IX-8- 13A); **B**, *H*. *floridana* (UF 24918). **C**, *T*. *mirus* gen. et sp. nov. **(**holotype, MLP 54-III-16-1); **D,** *H*. *septentrionalis* (HMNS 173); **E**, *H*. *paulacoutoi* (MCL-501/08) **F**, *P*. *humboldtii* (MCL-900/05). Abbreviations: **Gl**, Greatest length; **Wtt**, width at third trochanter; **Wde**, width at distal end.

The head of the femur is partially broken, presents a nearly circular contour interrupted by the *fovea capitis femoris* (a notch for the insertion of the round ligament) like in all Pampatheriidae. In *T*. *mirus*, the neck of the femur is less defined than in *Kraglievichia* (i.e., cf. *K*. *paranensis*).

In *T*. *mirus*, the lesser trochanter is longer than wide, recurved inside, sub-oval, and is more developed (Fig 8B) than in *P*. *humboldtii* (Fig 11I) and *H*. *paulacoutoi*, CTES-PZ 7495 (Colección Paleozoología de la Facultad de Ciencias Exactas y Naturales y Agrimensura, Universidad Nacional del Nordeste, Corrientes, Argentina). In *Kraglievichia* (i.e., cf. *K*. *paranensis*) (Fig 11B) and *H*. *floridana* (Fig 11D) is very small and caudally located.

The third trochanter of the Pampatheriidae is generally medially located to both ends, in the Propalaehoplophorinae (e.g. *Propalaehoplophorus australis*, MLP 16–15) occurs in a more distal position, whereas in other glyptodonts the third trochanter is fused to the epicondyles (e.g. *Neosclerocalyptus ornatus* neotype, MLP 16–28). In *T*. *mirus*, the third trochanter is very robust, broad and rough and in *Kraglievichia* cf. *K*. *paranensis*, *P*. *humboldtii* and *H*. *floridana* the outline is narrower and less rough.

The largest proportional width of both epicondyles (lateral and medial) is observed in *T*. *mirus* (Figs 9B and 10B), *S*. *antelucanus* (Fig 11F) and *Kraglievichia* (i.e., cf. *K*. *paranensis*) (Fig 11B), where transversely exceeds the condyles, whereas in *H*. *floridana* (Fig 11D) the epicondyles are poorly developed. In *T*. *mirus*, the medial condyle (internal) is much smaller than the lateral (external) one whereas in the remaining species, this difference in size is much smaller (Figs 9B and 10B). The intercondyloid fossa, between the condyles, is deeper, longer and wider than in *Kraglievichia* (i.e., cf. *K*. *paranensis*) and *H*. *floridana*, but less than in *P*. *humboldtii*.

The patellar facet (*trochlea femoris*) is distal and opposite to the condyles. In *T*. *mirus* is more asymmetric than in *S*. *antelucanus*, *Kraglievichia* (i.e., cf. *K*. *paranensis*) and *H*. *floridana*, but much less asymmetrical than in *P*. *humboldtii* and *H*. *paulacoutoi* (Fig 11A–11K).

The supra patellar fossa is above the patellar surface that in *T*. *mirus* is wider and deeper than in any other Pampatheriidae (Figs 9A and 10A).

**Patella**—this is the first record of a patella for a Pampatheriidae. The patella has a triangular morphology with rounded angles, a proximal base and a distal apex. The articular surface is posterior and the anterior surface is rounded (Figs 9C, 9D, 10C and 10D).

## Discussion

The osteoderms of Cingulata are the most commonly preserved elements that are mostly found isolated and/or disarticulated whereas large portions of caparace are rarely preserved. According to this, the systematic basis of the group was and is frequently built on the characters of these bone components [61, 23, 62, 63, 64, 65] although other phylogenetic approaches have exclusively used cranio-mandibular characters [29, 66]. The osteoderms of Pampatheriidae have a simpler ornamental pattern than other cingulates. Common structures (central figure, sulci and peripheral figures) for Glyptodontidae and Dasypodidae are absent in both Pampatheriidae and Peltephilidae [31, 67]. The main structure of the osteoderms of pampatheres is the longitudinal central elevation (not equivalent to the central figure of dasypodids and glyptodontids) [31].This elevation may have different morphologies, acute or keeled (e.g. *S*. *hondaense* and *S*. *carinatum* [31], high and rounded (e.g. *H*. *paulacoutoi* and *H*. *major*) [35] or flattened or diffuse (sometimes almost indistinguishable e.g. *P*. *typum*) [35].

The combination of morphological features of the osteoderms of *T*. *mirus* distinguishes from all species of the family:1) intermediate thickness between *Pampatherium* and *Holmesina*; 2) more denticulated sutural surface than in *P*. *typum* but less than in *H*. *major* and *H*. *paulacoutoi*; 3) very wide anterior and lateral margins, with several large and deep foramina; 4) wide and uniform marginal elevation in the fixed osteoderms and very narrow marginal elevation in movable and semi-movable osteoderms of the scapular and pelvic buckler; and 5) the longitudinal central elevation presents a general pattern, it is very asymmetric in each osteoderm, slightly keeled and with lateral circular or sub-circular projections.

Of all the characters mentioned on the morphology of the osteoderms of *T*. *mirus*, the most remarkable is the shape and design of the longitudinal central elevation. Based on the available evidence it is difficult to know, for the moment, the possible affinities of *T*. *mirus* with other Pleistocene Pampatheriidae (*Pampatherium* and *Holmesina*). However, the presence of a longitudinal central elevation flanked by two grooves is interpreted as a basal character for all Cingulata [35]. According to this, the presence of a conspicuous longitudinal central elevation flanked by longitudinal depressions in *Holmesina* could be considered the basal condition of all Pampatheriidae. This pattern is also present in older genera like *Kraglievichia* and *Scirrotherium*, although the central longitudinal elevation is more marked. In *Pampatherium* and *Vassallia* the longitudinal central elevation could represent a derived condition [35] as it is uniform and less sculpted. It could be considered that the complex ornamentation of the osteoderms of *Tonnicinctus* would be the result of a combination of characters present in *Pampatherium* and *Holmesina*. The longitudinal central elevation in *T*. *mirus* is lower and less delimited than in *Holmesina* but not as diffuse or flattened as in *Pampatherium*; additionally to the fact of having lateral projections that asymmetrically branch from each side of the longitudinal central elevation, which is an exclusive character, absent in all other Pampatheriidae.

The femur of *T*. *mirus* has a combination of morphology and size between the forms of the Neogene (*S*. *antelucanus Kraglievichia* (i.e., cf. *K*. *paranensis*) and *H*. *floridana*) and Quaternary taxa (*P*. *humboldtii* and *H*. *paulacoutoi*): 1) is more robust than in *Kraglievichia* (i.e., cf. *K*. *paranensis* and *H*. *floridana* but less robust than in *P*. *humboldtii* and *H*. *paulacoutoi*; 2) *Caput femoris* much less marked compared to other pampatheres; 3) great development of the greater trochanter ventral to the femoral head; 4) trochanteric fossa slightly marked, the third trochanter is very robust, broad, rugged and the proximal edge has a semicircular contour; 5) the lesser trochanter is large and more ventrally located than in other species; 6) the distal portion is more robust than other species and the width of the epicondyles ventrally surpasses the condyles.

Recognizing *Tonnicinctus mirus* gen. et sp. nov., increases the diversity of Pampatheriidae during the Pleistocene in North, Central and South America, consisting now in nine species grouped in three genera: 1) *Pampatherium* (*P*. *humboldtii*, *P*. *typum* and *P*. *mexicanum*); 2) *Holmesina*: (*H*. *septentrionalis*, *H*. *major*, *H*. *occidentalis*, *H*. *paulacoutoi* and *H*. *rondoniensis*); and 3) *Tonnicinctus* gen. nov. (*T*. *mirus* sp. nov.).

The Ensenadan Stage/Age (lower–middle Pleistocene, ca 2.0–0.4 Ma) [45] was characterized by a predominance of arid or semi–arid and colder climates than today, alternating with short periods of warmer and more humid conditions [68, 48, 43]. Probably the Ensenadan Stage/Age was the period in the Pleistocene with a great number of mammals with ecological requirements of more open and arid environments [69].The diversity of pampatheres during this Stage/Age is lower than that of the other cingulates, only two species of Pampatheriidae are recorded so far (*P*. *typum* and *T*. *mirus* gen. et sp. nov.) compared to six species of Dasypodidae (*Chaetophractus villosus* (Desmarest, 1804), *Ch*. *vellerosus* (Gray, 1865), *Zaedyus pichiy* (Desmarest, 1804), *Eutatus pascuali* Krmpotic, Carlini and Scillato-Yané, 2009, *Tolypeutes matacus* (Desmarest, 1804), and *Propraopus grandis* (Ameghino, 1881) and, at least, six species of Glyptodontidae (*G*. *munizi* Ameghino,1889, *Panochthus intermedius* Lydekker, 1894, *Pa*. *subintermedius* Castellanos, 1933, *Neuryurus rudis* (Gervais, 1878), *Neosclerocalyptus ornatus* (Owen, 1845) and *N*. *pseudornatus* (Ameghino, 1889) [41, 43].

The Lujanian Stage/Age (upper Pleistocene–lower Holocene; 0.13 to 0.008 Ma) was a period defined by the predominance of cold, arid and semi-arid climates, open environments, markedly lower average temperatures (that the currently recorded in the Pampean region), and with alternation of some brief pulses of humid and warmer conditions [68, 48, 70, 71]. The diversity of pampatheres during this Stage/Age is similar to that of the dasypodids and is lower than the Glyptodontidae. A total of five species of Pampatheriidae are recorded, two also present in the Ensenadan (*T*. *mirus* and *P*. *typum*) and three from the intertropical brasilic association and exclusive of the Lujanian (*P*. *humboldtii*, *H*. *paulacoutoi* and *H*. *major* (Lund, 1842)) [39, 35, 30, 72, 32]. While there are six species of Dasypodidae (*Chaetophractus villosus*, *Ch*. *vellerosus*, *Euphractus* sp. (Wagler, 1830), *Tolypeutes* sp. (Desmarest, 1804), *Propraopus grandis* and *Eutaus seguini* Gervais, 1867) and eight species of Glyptodontidae (*G*. *clavipes* Owen 1839, *G*. *reticulatus* Owen, 1845, *G*. *elongatus* Burmeister, 1866, *Pa*. *tuberculatus* (Owen, 1845), *Pa*. *frenzelianus* Ameghino, 1899, *D*. *clauvicaudatus* (Owen, 1847), *N*. *paskoensis* (Zurita, 2002) and *Ne*. *trabeculatus* Zurita and Ferrero, 2009) [73, 74, 75].

According to this, the diversity of the Pampatheriidae in Argentina is greater during the Lujanian Stage/Age (five spp.) than the Ensenadan Stage/Age (two spp.) and is similar to that of the Dasypodidae (six spp.) but lower than the diversitiy of the Glyptodontidae (eight spp.). This diversity of pampatheres of the Lujanian Stage/Age in Argentina is comparable to that recorded in the upper Pleistocene of Brazil, for which five species are also known (*P*. *typum*, *P*. *humboldtii*, *H*. *paulacoutoi*, *H*. *major* and *H*. *rondoniensis*) but grouped in two genera (three in Argentina) [35, 30, 72].

During the Lujanian Stage/Age, the pampatheres had a wide latitudinal distribution in Argentina, recorded in sediments of the provinces of Buenos Aires, Santa Fe, Corrientes, Entre Ríos, Santiago del Estero and Formosa [35, 30].

## Conclusions

*Tonnicinctus mirus* gen. et sp. nov. is recognized for the lower–middle Pleistocene (Ensenadan Stage/Age) and for the upper Pleistocene–early Holocene (Lujanian Stage/Age) of Buenos Aires and Santa Fe provinces. This taxon is the third genera and seventh species of Pleistocene Pampatheriidae for the of South America.

*Tonnicinctus mirus* gen. et sp. nov. has a complex ornamental pattern on its osteoderms which makes it particularly distinguishable from other species of Pampatheriidae, especially from the Pleistocene genera *Pampatherium* and *Holmesina*, whose ornamental patterns are simpler.

The pampatheres diversity during the Pleistocene is lower in the Ensenadan (two species) than in the Lujanian (five species).

## Appendix

Appendix 1. Specimens examined for comparative study.

### Pampatheriidae

*Scirrotherium hondaense*: UCMP 40056 (paratype), 37979, 38066, 38883.

*S*. *carinatum*: MLP 69-IX-8-13AB (holotype), 69-IX-8-13AC (paratype), 69-IX-8-13AD, (paratype), 52-X-1-35 (paratype), 69-IX-8-13AE (paratype), 70-XII-29-1 (paratype).

*S*. *antelucanus*: CFM-2867 (holotype) 355, 1639.

*Kraglievichia paranensis*: MLP 41-XII-13-903, 41-XII-13-911, 41-XII-13-912, 60-VI-18-68, 69-VIII-22-3, 76-VI-12-1.

*Kraglievichia* cf. *paranensis*: MLP 69-IX-8-13A.

*Vassallia minuta*: MLP 29-IV-15-6, 29-X-10-12, 29-X-8-39, 69-IX-5-21, 69-XII-26-17, 95-VIII-1-1.

*Plaina brocherense*: MUFyCA 769 (holotype).

*Pampatherium humboldtii*: MCL 900, 900/05, 900/06, 2308/01–798, MLP 81-X-30-1, MACN Pv 8490, 11905.

*P*. *typum*: MLP 34-IV- 12–6, 52-IX-28-20, 69-VIII-22-4, 69-VIII-25-11.

*P*. *mexicanum*: INAH 6201 (holotype).

*Holmesina septentrionalis*: AMNH 23435, 26856 (neotype), ROM 19787, 19790, UF 16372, HMNS 173.

*H*. *floridana*: UF 17476, 24918, 184326.

*H*. *occidentalis*: EPN, V. 1068 (paratype), 1176 (paratype), 1086 (paratype), 1103 (paratype), ROM 26121–26170.

*H*. *paulacoutoi*: MCL-501/01 (holotype), 501/08 (holotype), 501/86–103 (holotype), MLP 69-VIII-25-13, MACN Pv14400, CTES-PZ 7495.

*H*. *rondoniensis*: MERO-P-002 (holotype).

### Dasypodidae

*Dasypus puntactus*: MN 552-V

### Glyptodontidae

*Propalaehoplophorus australis*: MLP 16–15.

*Neosclerocalyptus ornatus*: MLP 16–28 (neotype).
